# Evans Blue Reduces Neuropathic Pain Behavior by Inhibiting Spinal ATP Release

**DOI:** 10.3390/ijms20184443

**Published:** 2019-09-09

**Authors:** Yuhua Yin, Jinpyo Hong, Thuỳ Linh Phạm, Juhee Shin, Do Hyeong Gwon, Hyeok Hee Kwon, Nara Shin, Hyo Jung Shin, Sun Yeul Lee, Won-hyung Lee, Dong Woon Kim

**Affiliations:** 1 Department of Medical Science, Chungnam National University School of Medicine, Daejeon 35015, Korea; 2 Department of Anesthesiology and Pain Medicine, Chungnam National University Hospital, Daejeon 35015, Korea; 3 Department of Anatomy, Brain Research Institute, Chungnam National University School of Medicine, Daejeon 35015, Korea

**Keywords:** Evans blue, neuropathic pain, spinal nerve ligation, vesicular nucleotide transporter, ATP, analgesic effect

## Abstract

Upon peripheral nerve injury, vesicular ATP is released from damaged primary afferent neurons. This extracellular ATP subsequently activates purinergic receptors of the spinal cord, which play a critical role in neuropathic pain. As an inhibitor of the vesicular nucleotide transporter (VNUT), Evans blue (EB) inhibits the vesicular storage and release of ATP in neurons. Thus, we tested whether EB could attenuate neuropathic pain behavior induced by spinal nerve ligation (SNL) in rats by targeting VNUT. An intrathecal injection of EB efficiently attenuated mechanical allodynia for five days in a dose-dependent manner and enhanced locomotive activity in an SNL rat model. Immunohistochemical analysis showed that EB was found in VNUT immunoreactivity on neurons in the dorsal root ganglion and the spinal dorsal horn. The level of ATP in cerebrospinal fluid in rats with SNL-induced neuropathic pain decreased upon administration of EB. Interestingly, EB blocked ATP release from neurons, but not glial cells in vitro. Eventually, the loss of ATP decreased microglial activity in the ipsilateral dorsal horn of the spinal cord, followed by a reduction in reactive oxygen species and proinflammatory mediators, such as interleukin (IL)-1β and IL-6. Finally, a similar analgesic effect of EB was demonstrated in rats with monoiodoacetate-induced osteoarthritis (OA) pain. Taken together, these data demonstrate that EB prevents ATP release in the spinal dorsal horn and reduces the ATP/purinergic receptor-induced activation of spinal microglia followed by a decline in algogenic substances, thereby relieving neuropathic pain in rats with SNL.

## 1. Introduction

Neuropathic pain is one of the most debilitating chronic pain syndromes, which can occur following nerve injury caused by various etiologies, such as diabetic neuropathy, post-herpetic neuralgia, drug-induced neuropathy, and traumatic nerve injury [1]. Most of these pathological conditions initially evoke the immune and inflammatory response in nervous tissue by the first activator molecules released from damaged afferent neurons [1,2]. These activators, known as neurotransmitters, subsequently bind to their corresponding receptors, located not only on the post-synaptic neurons, but also on the surface of microglia in the spinal cord, triggering the activation of these cells [2,3]. Neurons and glial cells interact to control the development and maintenance of neuropathic pain. Among various neurotransmitters, ATP is a prominent activator of microglia [4]. Following nerve injury, the increased ATP release from the damaged neurons through its purinergic receptors (exclusively expressed in spinal microglia) leads to the activation of multiple inflammatory signaling pathways potentiating neuroinflammation [4,5]. As ATP and its purinergic receptors are the keys to establishing mechanical allodynia, inhibiting ATP release from nerve terminals in the spinal cord may prevent the activation of inflammation-related molecules and subsequently alleviate the pain.

The vesicular nucleotide transporter (VNUT) is a member of the vesicular transporter family known as Slc17a9, a secretory vesicular protein responsible for the storage and release of ATP [6,7]. The phrase “VNUT-mediated (or dependent) ATP release” indicates that VNUT is required for the release of ATP within cells and has been used in many previous studies related to the function of ATP. In terms of neurological disease, it has been demonstrated that ATP is released from neurons in a VNUT-dependent manner, and knockdown of VNUT blocks the increased ATP level and reduces hypersensitivity following peripheral nerve injury [8]. These indicate that VNUT may be a target for preventing increased ATP level as well as the activation of purinergic receptors in the spinal cord, thereby relieving inflammation and pain [9].

Evans blue (EB) is an azo dye and has been commonly used in viability assays to penetrate non-viable cells, to estimate the proportion of body water in human blood, or to assess the integrity of the blood‒brain barrier due to its nontoxicity [10,11,12,13]. Notably, EB has been well known as an inhibitor of vesicular transporters including vesicular glutamate transporters (VGLUTs) [14,15], and vesicular nucleotide transporter (VNUT) [16]. It is possible that EB can change pain behaviors following nerve injury by inhibiting VNUT-mediated ATP release.

In the present study, we focused on addressing two major questions: whether EB could effectively reduce the ATP level in the spinal cord, subsequently relieving pain, and whether the mechanism by which EB inhibits VNUT activity and VNUT-mediated ATP release is involved in this effect. Although several previous studies have used EB to suppress ATP release, no report has revealed EB as a therapeutic treatment of neuropathic pain in a similar manner. Our study provides evidence for the possible therapeutic benefit of EB to treat neuropathic pain.

## 2. Results

### 2.1. SNL Induces Neuropathic Pain Behavior and Activates Microglia in the Ipsilateral Dorsal Horn of the Spinal Cord

**Figure 1:**
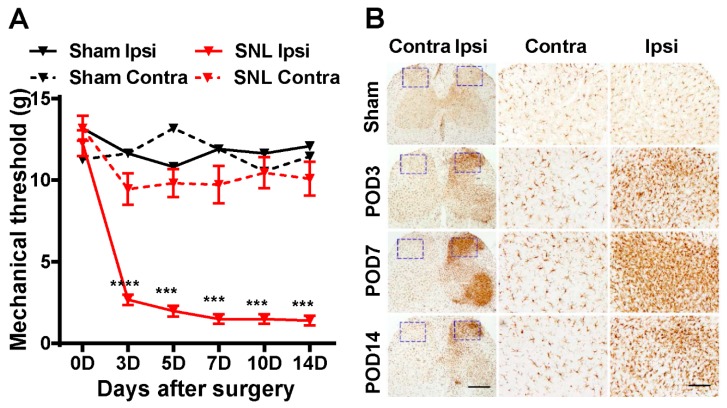
SNL induces neuropathic pain and activates spinal microglia in the ipsilateral dorsal horn. (**A**) Mechanical threshold was measured using the von Frey filament test on days 0 (baseline), 3, 5, 7, 10, and 14 after SNL surgery. Data are expressed as the mean ± SEM (one-way ANOVA with Dunnett’s post hoc test: *F*_(5, 60)_ = 102.6, *p* < 0.0001; *** *p*  <  0.001 vs. 0D Contra Ipsi; *n* = 11). (**B**) Spinal sections (L5) from the sham or SNL groups were immunostained with anti-Iba1 antibody, a specific microglial marker. The middle and right panels display the higher magnifications of the corresponding images in the purple dotted frames. Scale bars = 2 mm (**left panel**) and 400 μm (**middle and right panel**). Contra, contralateral; Ipsi, ipsilateral; POD, postoperative day.

To test the effect of EB on nerve injury-induced neuropathic pain, we first established a neuropathic pain model in rat by L5 spinal nerve ligation (SNL) [17]. Rats that passed a baseline von Frey filament test (≥10 g) underwent the surgery procedure. Then the von Frey filament test was repeatedly conducted to both groups of the rats: the sham and SNL groups on postoperative day (POD) 3, 5, 7, 10, and 14. Neuropathic pain was considered present when rats responded to a ≤4 g filament stimulus. The lower the mechanical threshold observed in the rats, the more sensitive to pain they are. In the SNL group, the mechanical threshold was early decreased (<4 g) on POD 3 on the ipsilateral (ipsi) side, compared with the contralateral (contra) side, and this effect was maintained up to POD 14 (Figure 1A). By contrast, no significant difference was detected between the ipsi and contra sides of the sham group; neither side showed hypersensitivity (Figure 1A). These results indicate that the SNL procedure we performed successfully induced neuropathic pain in the SNL rats.

We next examined the alteration of microglia in the spinal cord of the rats. The spinal cord sections on POD 3, 7, and 14 from both groups were immunostained with Iba1 (a specific microglial marker) (Figure 1B). In the sham group, the immunostaining sections do not display any significant changes between the ipsi and contra sides, and the microglial population was in normal condition. However, in the SNL group, as a result of peripheral nerve injury, the activation of microglia was detected in the ipsi spinal dorsal horn in all the sections of POD 3, 7, and 14. Of note, the section of POD 7 shows not only the maximum level of microgliosis reached on day 7 post-surgery, but also the activation of microglia in the ventral horn due to mirror-image allodynia, which often occurs when microglia are activated in the spinal cord [18]. Consistent with many previous studies, our results indicate the strongest activation of microglia peaked on day 7 post-surgery [4].

Taken together, these results demonstrate that we established a rat neuropathic pain SNL model showing pain behavior and microglial activity in the ipsilateral dorsal horn of the spinal cord.

### 2.2. EB Attenuates Pain Behavior and Enhances Locomotive Activity in SNL-Induced Rats

**Figure 2:**
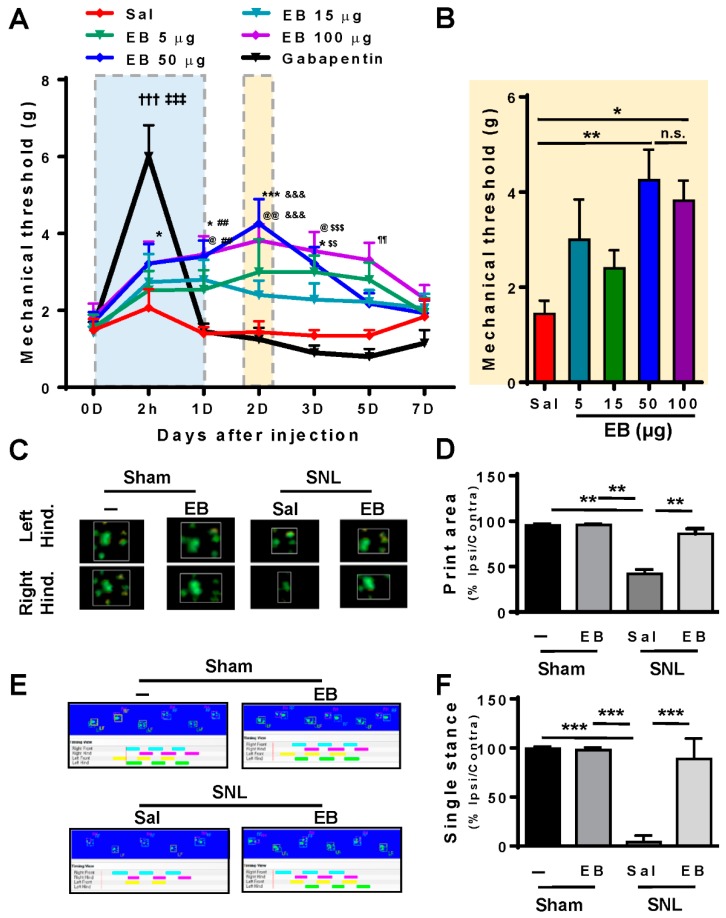
Intrathecal injection of EB attenuates pain behavior and enhances gait in rats with SNL-induced neuropathic pain. (**A**,**B**) On postoperative day 7, rats were randomly divided into six groups and each group was administered intrathecal treatment as follows: (1) saline, (2) EB 5 µg, (3) EB 15 µg, (4) EB 50 µg, (5) EB 100 µg in 10 μL saline, and (6) gabapentin 5 mg in 10 μL saline (*n* = 7–10 for groups 1–5; *n* = 4 for group 6). Then, the mechanical threshold was determined on day 0 (baseline), 2 h, and 1, 2, 3, 5, and 7 days after injection. Data are expressed as the mean ± SEM (two-way ANOVA with Dunnett’s post hoc test for each reagent, *F*_(6, 354)_ = 8.385, *p* < 0.0001: * *p* = 0.0461 (2 h), * *p* = 0.0192 (1D), *** *p* < 0.0001 (2D) and * *p* = 0.0461 (3D) vs. EB 50 µg 0D, ^@^ *p* = 0.0276 (1D), ^@@^ *p* = 0039 (2D), and ^@^ *p* = 0.0175 (3D) vs. 100 µg 0D, ^†††^ *p* < 0.0001 vs. gabapentin 0D; two-way ANOVA with Dunnett’s post hoc test for each day, *F*_(5, 354)_ = 11.90, *p* < 0.0001: ^‡‡‡^ *p* < 0.0001 (gabapentin) vs. Sal 2 h, ^##^ *p* = 0.0033 (EB 50 µg) and ^##^ *p* = 0.0024 (EB 100 µg) vs. Sal 1D, ^&&&^ *p* < 0.0001 (EB 50 µg) and ^&&&^ *p* = 0.0003 (EB 100 µg) vs. Sal 2D, ^$$^ *p* = 0.0068 (EB 50 µg) and ^$$$^ *p* = 0.0010 (EB 100 µg) vs. Sal 3D, ^¶¶^ *p* = 0.0041 (EB 100 µg) vs. Sal 5D. Data for gabapentin, saline, and EB treatment at the time points of 2 hours and day 1 following injection were marked (blue box). Data from day 2 following EB injection were further analyzed (yellow box). Data are expressed as the mean ± SEM (one-way ANOVA with Dunnett’s post hoc test, *F*_(4, 43)_ = 4.3888, *p* = 0.0048, ** *p* = 0.0015 (EB 50 µg) and * *p* = 0.0191 (EB 100 µg) vs. Sal 2D; n.s). (**C**–**F**) On postoperative day 7, animals were divided into four groups (*n* = 6 per group) and administered either saline or EB (EB 50 µg) intrathecally. After two days, the print area (**C**,**D**) and single stance (**E**,**F**) were measured using the catwalk gait system for the Contra and Ipsi hind paws, and expressed as the percent of Ipsi/Contra. Data are expressed as the mean ± SEM (unpaired Student’s *t*-test: print area (*t*_(4)_ = 10), *** *p* < 0.0001 vs. Sal; single stance (*t*_(4)_ = 6.639), ** *p* = 0.0027 vs. Sal). –, non-treated; EB, Evans blue; Sal, saline; Hind., hind paw; n.s., non-significant.

To clarify the concept that EB could impair the SNL-induced neuropathic pain, we administered EB into the spinal cords of the SNL rats. EB was injected intrathecally in the spinal cords of rats with different doses of 5 (27.75 µg/kg body weight), 15 (83.25 µg/kg), 50 (277.5 µg/kg), or 100 µg (555 µg/kg) per rat. We also applied 5 mg (27.75 mg/kg) of gabapentin, a drug prescribed clinically for the treatment of neuropathic pain [19], to the SNL rats to compare the analgesic effect with the EB treatment. In addition, the SNL rats treated with saline were used as the control group. The injection procedure was conducted to SNL rats on POD 7 when the microglial activation peaked up. Following the injection, the pain behavioral tests were administered to rats in all treatment groups to determine the analgesic effect of each dose of EB, compared with gabapentin and saline treatments (Figure 2). Figure 2A shows the results obtained in von Frey tests that were carried out at selected time points: at 2 h, 1, 2, 3, 5, 7 days post-injection. The SNL saline-treated rats did not show any improvement on the mechanical threshold, while the SNL gabapentin-treated rats showed the greatest pain relief 2 h post-injection and it diminished within several hours. Interestingly, the treatment of EB brought about an analgesic effect in a dose-dependent manner. For all doses applied, the mechanical thresholds of the rats were increased, compared to saline-treated rats. This effect was maintained for almost five days post-injection and the greatest effect was recognized on day 2 post-injection with the dose of 50 µg EB. We also have quantitative data (Figure 2B) comparing the mechanical thresholds of the SNL rats treated with different doses of EB. The data suggest that 50 µg was the most effective dose. A higher dose of 100 µg did not bring about better pain relief.

We then conducted the catwalk gait test, another sign of pain in SNL-treated rats (Figure 2C,D) [20]. In this assay, rats were randomly separated into four groups: the sham, the sham EB-treated group, the SNL saline-treated, and SNL EB (50 µg)-treated groups. Based on the results of the von Frey test, the catwalk test was performed on day 2 post-injection because the treatment of 50 µg EB reached the greatest effect. Figure 2C shows the representative images of the foot print areas of the right and left hind paws (contra and ipsi sides), illustrating the complete surface area of the hind paw once it touched the glass plate. In the sham group and the sham EB-treated group, we did not observe any significant difference in the print areas between the ipsi and contra hind paws. However, in the SNL saline-treated group, the print area of the ipsi high paw was much reduced in length and width, compared with the contra side. In the SNL EB-treated group, the print area of the left hind paw was enhanced, so the difference between the ipsi and contra sides was not significant. The quantitative data (Figure 2D) show the proportion of ipsi/contra foot print area, suggesting ratios of 42.0%, 86.02%, 100%, and almost 100% in the SNL saline-treated, SNL EB-treated, sham, and sham EB-treated groups, respectively. The print area was improved by 55% in the SNL EB-treated group, compared with the SNL saline-treated

The second parameter we measured in the catwalk test was the single stance assessed by the duration of a single hind paw touching the glass plate (Figure 2E,F). The sham and sham EB-treated rats displayed a normal single stance and the proportion of ipsi/contra was almost 100%. In the SNL EB-treated group, this proportion was over 80%. However, in the SNL saline-treated group, it was lower than 5%. The image analysis (Figure 2E) and the quantitative data (Figure 2F) display an increased ratio of ipsi/contra in the SNL EB-treated group by 80%, compared with the SNL saline-treated group. These results could be explained by the pain reduction in the ipsi high paw of the rats treated with EB; thus, locomotive activity was improved dramatically in this group.

Collectively, these results indicate that EB alleviates neuropathic pain behavior and improves gait in rats with SNL-induced neuropathic pain. The normal locomotive activity observed in the sham and the sham EB-treated groups demonstrates that EB has no effect on rats without neuropathic pain.

### 2.3. EB Reduces the Level of ROS and Proinflammatory Mediators by Downregulating Microglial Activity in the Spinal Dorsal Horn

**Figure 3:**
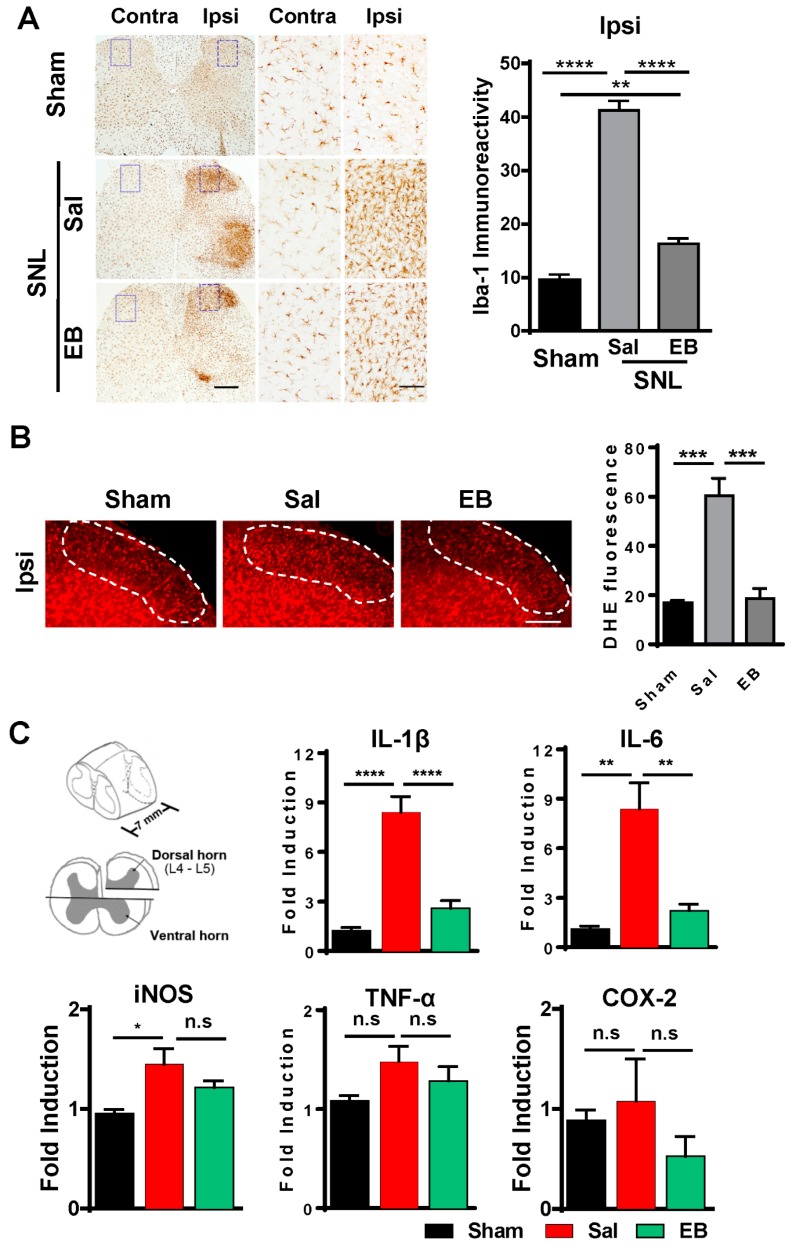
EB reduces SNL-induced microglial activation and decreases the generation of ROS and the gene expression of proinflammatory mediators in the ipsilateral dorsal horn of the spinal cord in rats with SNL-induced neuropathic pain. (**A**) Saline or EB (50 µg) were introduced by intrathecal injection on postoperative day 7. After two days, spinal cord sections (L5) were prepared and immunostained with anti-Iba1 antibody. The middle and right panels display the higher magnifications of the corresponding images in the purple dotted frames. Scale bars = 2 mm (**left panel**) and 400 µm (**middle and right panel**). Microglial reactivity was then measured in Iba1-immunoreactive cells using ImageJ software (version 1.50i. Java 1.6.0). Data are expressed as the mean ± SEM (one-way ANOVA with Tukey’s post hoc test, ** *p* < 0.01, **** *p* < 0.0001 vs. Sal; *n* = 3 per group). (**B**) Saline or EB (50 µg) were administered intrathecally on postoperative day 7. After two days, spinal sections (L5) of the sham, the SNL saline-treated and EB-treated rats were incubated with DHE (1 μM) for 5 min. The laminae I, II of the spinal dorsal horn are marked with white dotted frame. Scale bars = 200 µm (**left panel**) and 500 µm (**right panel**). The levels of ROS in the tissues were later obtained by measuring the intensity of DHE fluorescence using ImageJ software. Data are expressed as the mean ± SEM (unpaired Student’s *t*-test, *t*_(4)_ = 5.636, *** *p* < 0.001 vs. Sal; *n* = 6 per group). (**C**) Saline or EB (50 µg) were introduced by intrathecal injection on postoperative day 7. After two days, the L4–5 fragments of the ipsilateral dorsal horn were prepared and used for total RNA isolation. The levels of proinflammatory mediators, including IL-1β, IL-6, TNF-α, iNOS, and COX-2, were evaluated with real-time qPCR. Sham was used as a control. Data are expressed as the mean ± SEM (one-way ANOVA with Tukey’s post hoc test, *n* = 5–7 per group: iNOS, * *p* < 0.05 vs. Sal; IL-1β, **** *p* < 0.0001 vs. Sal; IL-6, ** *p* < 0.01 vs. Sal). DHE, dihydroethidium; IL, interleukin; TNF, tumor necrosis factor; iNOS, inducible nitric oxide.

In the pathogenesis of neuropathic pain, microglial activation, in response to neuronal activity, plays key roles in the induction, development, and maintenance of pain [4]. Treatment with EB indeed improved the outcome of pain behavioral symptoms in the SNL-induced rats. We next investigated whether the pain reduction in the EB-treated rats involved microglial activity. Spinal cord sections were collected from the three groups of rats, the sham, saline-treated, and EB (50 µg)-treated groups, on day 2 post-injection, and were subsequently immunostained with Iba1 antibody. Figure 3A shows the immunostaining images and the intensities of Iba-1 immunoreactivity in the spinal cords of the rats from the three groups. The images display stronger activation of microglia in the SNL saline-treated group compared to the EB-treated group, in which microglial activation did not increase much compared with the sham group. In detail, the intensity of Iba1 immunoreactivity was reduced by 57% in the spinal dorsal horn (SDH) of EB-treated rats (saline vs. EB, 38.22 ± 1.33 vs. 16.33 ± 0.55).

Microglia in response to neuronal activity control the inflammatory condition in the spinal cord by releasing inflammatory cytokines, thereby determining the pain state [4]. Following confirmation of microglial activation-related pain reduction in EB-treated group, we checked and compared the production and expression of proinflammatory mediators, including ROS, iNOS, IL-1β, IL-6, TNF-α, and COX-2 in the SDH, among the three groups of rats: the sham, SNL saline-treated, and SNL EB-treated groups. ROS describes a number of reactive molecules and free radicals derived from molecular oxygen that are generated during mitochondrial oxidative metabolism [21,22]. The more ROS is produced in the spinal cord, the more severe the inflammatory state is. To examine the production of this oxidative stress, we immunostained the spinal cord sections of the sham, SNL saline-treated, and SNL EB-treated rats with DHE (a ROS marker) on day 2 post-injection (Figure 3B). In a comparison between the sham and SNL saline-treated groups, ROS production was much more robust in the SNL saline-treated group, indicating the intense inflammatory state induced in the SDH of the SNL rats following surgery. However, in the SNL EB-treated group, a lower production of ROS was detected compared to the SNL saline-treated group. The reduction in the intensity of DHE fluorescence was 70% from the EB group (18.56 ± 2.39) to the saline group (60.43 ± 7.03).

We also checked the expression of proinflammatory cytokines in the SDH by quantitative real-time PCR (qRT PCR) (Figure 3C). Total mRNA was isolated from the SDHs of rats from each group on day 2 post-injection and analyzed for gene expression. As a result of microglial activation, the expression of proinflammatory cytokines including IL-1β, IL-6, iNOS, TNF-α, and COX-2 was upregulated in the SDH of the SNL saline-treated rats, compared with the sham rats [1], Although the difference in the regulation of iNOS, TNF-α, and COX-2 was not significant among the three groups, it was at least consistent with the regulation of IL-1β and IL-6. Interestingly, EB-reduced microglial activation resulted in the decreased expression of these mediators in the SDH of EB-treated rats. Of note, the mRNA levels of IL-1β and IL-6 were dramatically downregulated by 70% (IL-1β, 8.40 ± 0.96 vs. 2.59 ± 0.47), and 73% (IL-6, 8.37 ± 1.58 vs. 2.22 ± 0.40).

Taken together, these data suggest that EB attenuated pain behaviors by inhibiting microglial activation, followed by a decreased release of ROS and proinflammatory cytokines in the spinal cords of the EB-treated rats.

### 2.4. EB Blocks ATP Release from Neurons of the DRG and Spinal Dorsal Horn in a VNUT-Dependent Manner

**Figure 4:**
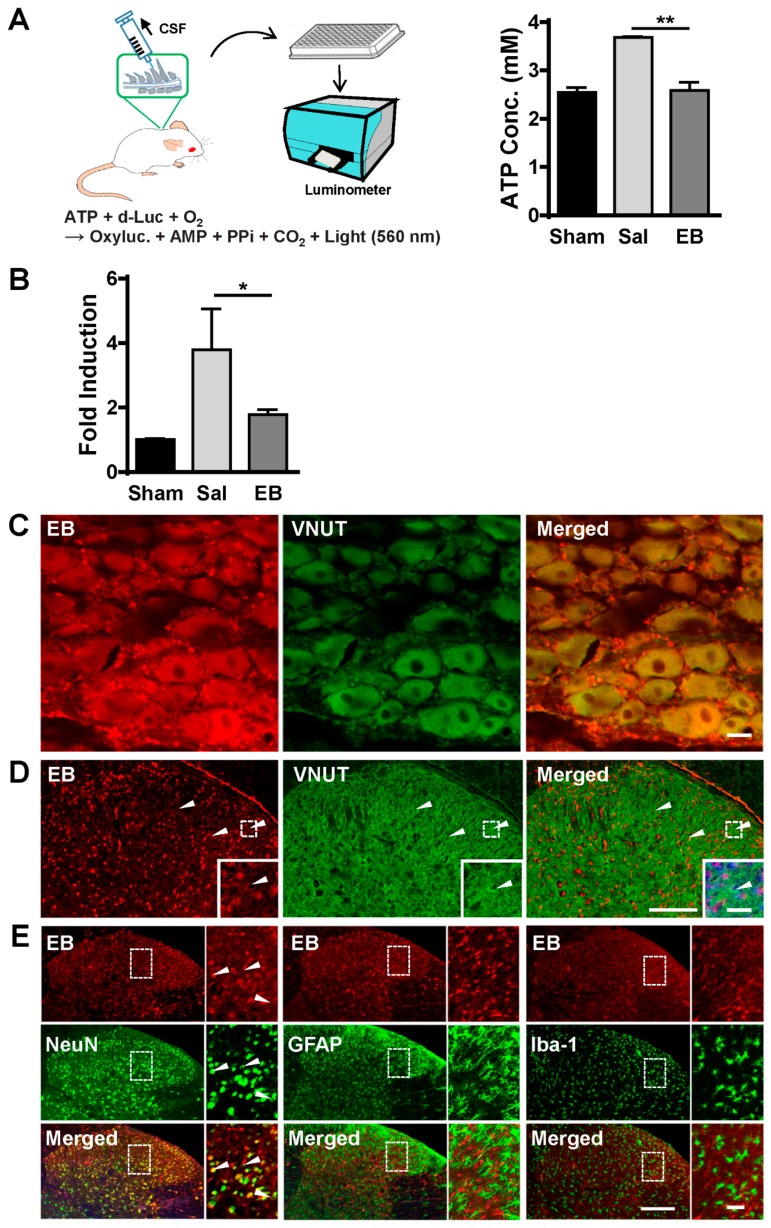
EB inhibits ATP release from neurons of the DRG and spinal cord of the SNL-induced rats in a VNUT-dependent manner. (**A**) Saline or EB (50 µg) were administered into the subarachnoid space by intrathecal injection on postoperative day 7. After two days, CSF was obtained from the cisterna magna and used to determine ATP levels. Sham used as a control. Data are expressed as the mean ± SEM (one-way ANOVA with Tukey’s post hoc test, *F*_(2, 6)_ = 31.80, *p* = 0.0006, ** *p* = 0.0012 vs. sham; *n* = 3 per group). (**B**) Saline or EB (50 µg) were administered by intrathecal injection on postoperative day 7. After two days, the SDHs were collected from rats and prepared for analysis of VNUT gene expression by qPCR. Data are expressed as the mean ± SEM (one-way ANOVA with Tukey’s post hoc test, * *p* < 0.05 vs. sham; *n* = 3 per group). (**C**) EB (50 µg) was administered by intrathecal injection on postoperative day 7. After two days, DRG tissues were immunostained with anti-VNUT antibody. White arrowhead, C-fibers; yellow arrowhead, Aδ fibers; blue arrowhead, Aβ fibers. Scale bar = 200 µm. (**D**) The spinal tissues from Figure 4C were also immunostained with anti-VNUT antibody. Arrowheads indicate EB-targeted neurons expressing VNUT. (**E**) The same tissues used in Figure 4D were immunostained with anti-NeuN (neuronal marker), anti-GFAP (astrocytic marker), or anti-Iba1 (microglial marker) antibodies. (**D**,**E**) The smaller images (on the right of each image) display the higher magnifications of the corresponding images in the white dotted frames. All scale bars = 200 µm.

Following evidence regarding the analgesic effect of EB on SNL-induced rats, we investigated the mechanism by which EB could relieve pain and improve pain behavior. Since EB has been well documented as a pharmacological inhibitor of vesicular transporter VNUT, which mediates the storage and release of ATP, a key activator of microglial activation in the spinal cord [23], we questioned whether the effect of EB on pain reduction was associated with this mechanism. To test this concept, first we checked the ATP level in the CSF. The CSF was collected directly from the cisterna magna of rats on day 2 post-injection. Figure 4A illustrates the ATP measurement process. The concentration of ATP in the CSF of the SNL saline-treated rats was 42% higher than that in the sham rats, whereas, in the EB-treated group, the ATP level was completely restored to the baseline level of the sham group (shown in the graph). The decreased level of ATP serves as evidence that EB may suppress microglial activation by blocking ATP release in the spinal cord of the SNL EB-treated rats.

As mentioned, extracellular ATP release from cells is mediated by VNUT [6,7,24]. The decreased ATP level in the CSF means VNUT activity may have also decreased in the spinal cord of EB-treated rat. Hence, we checked regulation of VNUT in the SDH of the rats following saline/EB treatment by qRT PCR (Figure 4B). As expected, the expression of VNUT was upregulated in the SNL saline-treated group, compared to the sham group. However, EB, as a VNUT inhibitor, prevented the upregulation of VNUT in the SDH of the EB-treated rats following the SNL procedure (Figure 4B). This led to a decreased ATP level in the CSF of the EB-treated group and correlates with the identification that EB is an inhibitor of VNUT-dependent ATP release [16].

We then tested the expression of VNUT in relation to EB. VNUT has been identified in a number of different cell types including biliary epithelia cells, T lymphocytes, pancreatic β cells, and particularly in various cell types of the nervous system [25,26,27]. VNUT has been shown to mediate ATP uptake and release in DRG neurons [23], dopaminergic neurons in the retina and midbrain [28], dorsal horn neurons, and glial cells [8,29,30]. It is likely that VNUT is widely expressed in nervous tissue. Since primary afferent neurons of the DRG, dorsal horn neurons, and glial cells have been demonstrated to play similarly crucial roles in the pathogenesis of neuropathic pain [4], we attempted to examine the expression of VNUT in these areas in relation to EB. The sections of the DRG and spinal cords of the EB-treated rats on day 2 post-injection were immunostained with an anti-VNUT antibody (Figure 4C,D). A study has reported that VNUT is found in all cell types of the DRG, including Aβ (large fiber), Aδ (medium fiber), and C (small fiber) [31]. While C-fiber activation is sufficient to elicit spinal microglial activation, activation of A-fibers is also important for maintaining microglial activation [32,33]. Consistently, our immunostaining results show that VNUT was expressed in all three cell types of the DRG of the EB-treated rat: Aβ, Aδ, and C (Figure 4C). Furthermore, in the spinal cord, VNUT immunoactivity was also observed ubiquitously in neurons and glial cells of the SDH, which correlates with the identification in previous studies [8] (Figure 4D). EB is an azo dye widely used in immunofluorescence counterstains. It can be visualized under fluorescence microscopy without any other fluorescent dyes (excitation at 620 nm, emission at 680 nm) in cryocut tissue. However, EB fluorescence in cryocut tissue does not last long. We attempted to process the staining procedure as soon as the tissues were removed from rats. However, it seems that the cryocut section and staining procedures weakened the intensity of EB in the stained sections. In spite of that, our staining results show that EB was found close to or overlapped with VNUT immunoactivity in both the DRG and SDH (Figure 4C,D). This indicates that EB could be in close interaction with VNUT in these cells. Moreover, to verify with which cell types EB mainly co-localized in the SDH, the spinal sections were immunostained with anti-Iba-1 (microglial marker), anti-GFAP (astrocytic marker), and anti-NeuN (neuronal marker) antibodies (Figure 4E). Interestingly, the staining data show that EB mainly co-localized with neurons (96.6%, left panel), but not with astrocytes (middle panel), or microglia (right panel). Collectively, these data suggest that EB may affect VNUT activity in neurons of either the DRG or SDH, but not glial cells. This effect resulted in decreased VNUT-dependent ATP release from neurons, thereby inhibiting microglial activation and pain state in the EB-treated rats.

### 2.5. EB Inhibits ATP Release from Cultured Neurons, but Not from Cultured Microglia or Astrocytes

**Figure 5:**
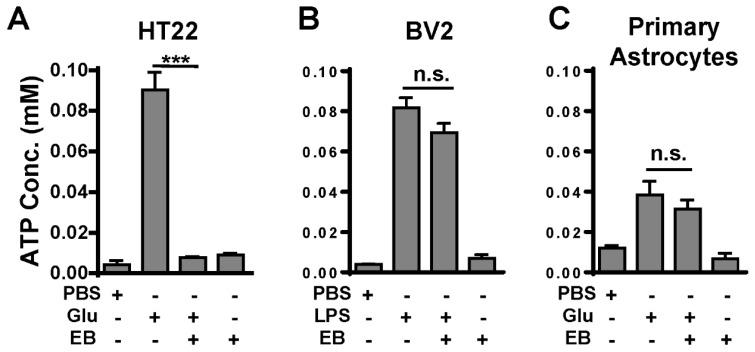
EB blocks ATP release from cultured neurons, but not from cultured microglia or astrocytes. (**A–C**) To investigate whether EB was able to disturb ATP release from neurons or glia, glutamate-induced ATP release from HT22 cells (mouse neuronal cell line) (**A**), LPS-induced ATP release from BV2 cells (**B**), and glutamate-induced ATP release from rat primary astrocytes (**C**) were determined in the presence or absence of EB for 2 h (EB, 1 mM; glutamate, 100 µM; LPS, 100 ng/mL). Data are presented as the mean ± SEM (one-way ANOVA with Tukey’s post hoc test, HT22 (*F*_(3, 4)_ = 180.1, *p* < 0.0001), *** *p* = 0.0002 vs. glutamate; BV2 (*F*_(3, 4)_ = 135.0, *p* = 0.0002), n.s; primary astrocytes (*F*_(6, 63)_ = 24.98, *p* = 0.0047), n.s.). **−**, non-treated; **+**, treated; LPS, lipopolysaccharide; CSF, cerebrospinal fluid; Glu, glutamate; PBS, phosphate-buffered saline.

The immunostaining results showed that EB was observed mainly in neurons, accompanied by evidence that EB blocked the increased ATP release level. The question arose of whether ATP release from glial cells is not truly influenced by EB. To address this question, we tested the effect of EB on cultured neurons, microglia, and astrocytes in vitro (Figure 5). HT22 (neuronal cell line), BV2 (microglial cell line), and primary astrocytes were cultured and stimulated by glutamate or LPS with/without incubation with EB prior to stimulus. As a result of activation, the levels of ATP release increased in all groups of the cultured cells (which were not incubated with EB), following glutamate/ LPS stimulus. However, in the group of glutamate-treated HT22, which had been incubated with EB prior to stimulus, the ATP release level was restored to the baseline as in the control groups (Figure 5A). ATP release from BV2 or astrocytes was not affected by EB treatment (Figure 5B,C). There was no significant difference in the levels of ATP release between EB-treated and non-treated groups of cultured microglia and astrocytes. These results indicate that EB may block ATP release from neurons, but not from microglia or astrocytes.

### 2.6. EB Significantly Alleviates Monoiodoacetate (MIA)-Induced OA Pain

**Figure 6:**
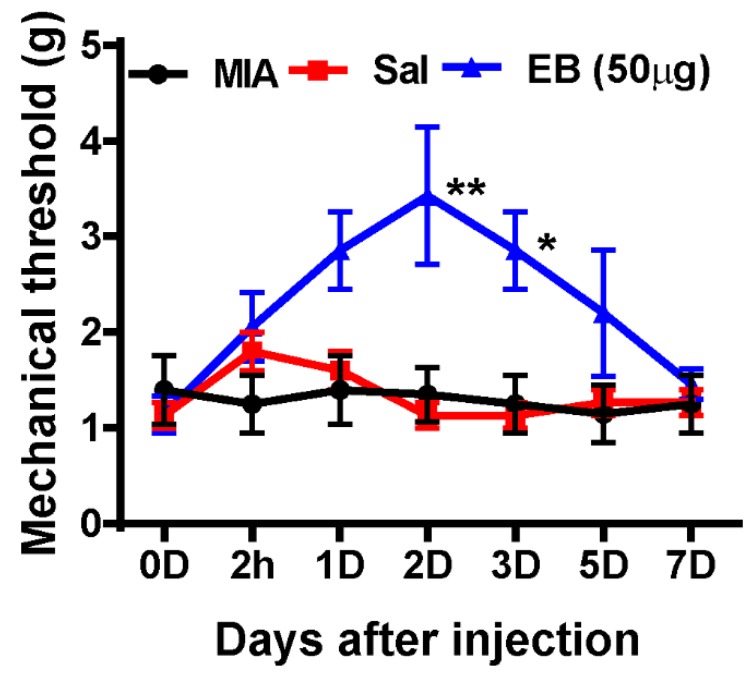
MIA-induced OA pain is effectively relieved by a single intrathecal introduction of EB. On MIA post-injection day 7 (2 mg in 25 μL), animals were separated into three groups, and each treatment was delivered intrathecally (no treatment, saline, EB 50 µg; *n* = 3–7 per group). The mechanical threshold was then evaluated at day 0 (baseline), 2 h, and days 1, 2, 3, 5, and 7 after injection. Data are expressed as the mean ± SEM (two-way ANOVA with Dunnett’s post hoc test: *F*_(2, 77)_ = 12.71, *p* < 0.0001, ** *p*  =  0.0024 vs. Sal 2D, * *p* = 0.0292 vs. Sal 3D). MIA, monoiodoacetate; OA, osteoarthritis.

For more evidence of the analgesic effects of EB, we finally attempted to test the influence of EB on a more physiologically relevant disease: osteoarthritis (OA). According to clinical surveys, neuropathic pain-like symptoms are present in a subpopulation (~30%) of patients with OA [34,35]. To establish an OA model in rats, MIA (2 mg in 25 µL saline) was intra-articularly administered to a hind leg, which gradually leads to OA due to the death of chondrocytes in the knee joint cartilage, which is attributed to the inhibition of ATP synthesis in the glycolytic pathway [36]. A similar injection procedure of saline/EB treatment to what was used in the SNL rat model was applied to MIA-induced rats on POD 7. The mechanical thresholds were then determined using von Frey filaments at the same time points of 2 h and 1, 2, 3, 5, and 7 days post-injection (Figure 6). Obviously, MIA induced pain in rats, expressed by the low mechanical thresholds in the MIA-induced group. Notably, the mechanical thresholds of the rats in the MIA EB-treated group were upregulated at 2 h and maintained up to day 5 post-injection. The most significant response was recognized on day 2 post-injection (Figure 6). In contrast, the saline-treated rats did not show any remarkable difference in pain behaviors compared to the MIA-induced rats.

These data correlate with the results obtained in the SNL-induced neuropathic pain model, demonstrating the analgesic effect of EB on different models of neuropathic pain.

## 3. Discussion

ATP, which is established as a source of free energy involved in biochemical pathways, has now been recognized as a crucial neurotransmitter, regulating the activation of purinergic receptors P2X and P2Y expressed in post-synaptic neurons, and on the surface of microglia and astrocytes [4,37,38]. This is an essential step, promoting microglial activation in the spinal cord and subsequently contributing to the development of neuropathic hypersensitivity. Along with evidence regarding the role of an increased extracellular ATP level to trigger neuropathic pain, the mechanism of ATP action has recently been discovered by Tsuda and colleagues [8]. They have demonstrated that VNUT and VNUT-mediated ATP release are involved in the induction and maintenance of pain. Their data also show that knockdown of VNUT gene could decrease ATP release and attenuate peripheral nerve injury-induced pain hypersensitivity. It has come to our attention that the pharmacological inhibition of VNUT could achieve a similar effect in terms of pain reduction. EB has long been known as pharmacological inhibitor of several vesicular transporters, including VGLUTs [14] and particularly VNUT [16]. In a previous study on diabetes, EB was used to suppress VNUT-mediated ATP release, followed by lower glucose and insulin secretion [39,40]. However, no study has reported the therapeutic benefit of EB in reducing neuropathic pain or neurological diseases.

It is reasonable to assume that EB can attenuate pain behaviors by inhibiting VNUT-dependent ATP release. As expected, all results obtained in pain behavioral tests support our hypothesis. All the doses (5, 15, 50, or 100 µg EB) that were applied to the SNL rats effectively enhanced the mechanical thresholds. Notably, the greatest consequence was achieved with the dose of 50 µg on day 2 post-injection. That was why most of our analyses were performed on SNL rats treated with 50 µg EB 2 days post-injection. It is also important to show that the toxicity of EB was previously tested in monkeys [11]. While intravenous injections of 50, 100, and 200 mg/kg of EB resulted in the death of the monkeys after 11, 7, and four days, respectively, post-injection of EB, an intravenous injection of 25 mg/kg of EB, was nonlethal to all monkeys. Further, the blue coloring of the skin and mucous membranes of the mouth disappeared after five weeks following EB treatment. Therefore, the dose of EB in our study was safe, as only 50 μg (277.5 μg/kg, body weight) of EB was administered intrathecally in rats with SNL to treat neuropathic pain (Figure 2A).

We further examined the mechanism by which EB could reduce pain in SNL rats. It has been well documented that EB is a pharmacological inhibitor of VNUT; we questioned whether this mechanism was directly associated with the analgesic effect of EB. The decreased ATP level and VNUT expression in EB-treated rats and the results of immunostaining likely address this question. EB has been used in previous studies as a VNUT inhibitor to suppress ATP release. Our data show that EB completely restored the ATP level to the baseline by preventing VNUT upregulation after the SNL procedure, and EB was found in VNUT immunoactivity. Although our data do not directly demonstrate that EB targeted VNUT, it is sufficient to prove that EB reduced pain by inhibiting VNUT-dependent ATP release. In fact, VNUT was expressed in both the DRG and spinal cord of the SNL EB-treated rats following nerve injury. However, EB was only observed to be close to or overlapped with VNUT in the neurons of either the DRG or spinal cord, not in the microglia or astrocytes. Our in vitro experiment, in which neurons, microglia, and astrocytes were cultured and then treated with a stimulus, also confirms that EB reduced ATP release from cultured neurons, but not from cultured microglia or astrocytes. In addition to neurons, microglia and astrocytes also have potential to express VNUT and release ATP [29,30]. Tsuda and colleagues have shown that VNUT and VNUT-dependent ATP release from SDH neurons are required for allodynia, and that peripheral nerve injury-induced neuropathic pain does not require VNUT in DRG neurons or glial cells [8]. Concomitantly, our data show that EB mainly targeted neurons in both the DRG and SDH. To the best of our knowledge, ATP is a prominent activator of microglia released from damaged afferent neurons, which provokes an inflammatory responses in the spinal cord, followed by the development of hypersensitivity [4]. Although they have shown that VNUT in DRG neurons does not contribute to the formation of neuropathic pain, somehow or other, ATP release from primary afferents, in response to nerve injury, promotes ongoing pain by activating purinergic receptors P2X and P2Y on post-synaptic neurons and on the surface of surrounding microglia in the spinal cord. This step is important, leading to settings of neuropathic pain in early stage. Subsequently, the ongoing pain requires not only neuronal activity but also microglial activation in interaction with neurons in the spinal cord. In addition to decreased ATP levels, our data also show weaker microglial activation in the SDH of the EB-treated rats compared to the saline-treated rats. Less robust microglial activation resulted from decreased ATP release and was followed by decreased levels of pro-inflammatory cytokines released from activated microglia. Taken together, the analgesic effect of EB on SNL-induced rats was performed via a mechanism by which EB inhibited VNUT-mediated ATP release from neurons in the DRG and SDH, followed by a decreased state of microglial activation-related neuroinflammation.

A limitation of this study is that we did not conduct a further investigation to find the mechanism of action of EB in the DRG neurons. EB is an inhibitor of not only VNUT, but also VGLUTs and the AMPA/kainite receptor [41,42]. The question of whether these other action mechanisms could be involved in the effect of EB on reducing the ATP level in CSF remains unclear. One point for discussion is that the analgesic effect of EB lasted up to five days post-injection, while a higher dose of gabapentin (5 mg) led to reductions in pain for only 2 h. Currently, several types of drugs, including nonsteroidal anti-inflammatory drugs (e.g., naproxen, ibuprofen), ion channel blockers (e.g., lidocaine, gabapentin, pregabalin), and opioids, are clinically prescribed for the treatment of neuropathic pain [43,44]. These drugs mainly act to downregulate synaptic plasticity and neuronal activity, and so modulate the consolidation and maintenance of pain. However, partially due to the short half-life of these drugs, the analgesic effects often last only several hours, whereas a lower dose (50 µg) of EB led to pain reduction for up to five days. An additional experiment we conducted on rats with the OA model reveals the analgesic effect of EB lasting for five days following injection. This long effect may be partially due to the administration of EB via intrathecal injection, or the longer half-life of EB in the spinal cord, or the multiple action mechanisms of EB in reducing pain. These questions require further investigation.

In conclusion, the aim of this study was to investigate whether EB can attenuate pain behaviors in neuropathic pain. EB has been indicated as a pharmacological inhibitor of VNUT, VGLUTs, and the AMPA/kainite receptor. All of these action mechanisms could be involved in the effect of EB on reducing pain. In the present study, we only attempted to investigate the analgesic effect of EB in its role as a VNUT inhibitor, suppressing ATP release. Our study not only suggests that EB could be a new therapeutic agent for the treatment of neuropathic pain, but also provides evidence for the involvement of VNUT and VNUT-dependent ATP release in the analgesic effect of EB. The questions regarding the other molecular mechanisms of the action of EB are important to address in future studies.

## 4. Materials and Methods

### 4.1. Experimental Animals

Male Sprague-Dawley rats (180–200 g) used in this study were obtained from Daehan Bio Link (DBL, Chung-buk, Korea). Animals were housed individually in cages under a controlled 12:12 h light: dark cycle at 23 °C. The rats were kept at least seven days under these conditions before surgery. Water and food were available ad libitum until the rats were transported to the laboratory approximately 1 h prior to the experiments. All experiments were carried out with the approval of the Animal Care and Use Committee at the Chungnam National University (CNUH-017-A0045, 26 December 2017) and were in accordance with the ethical guidelines of the National Institutes of Health and the International Association for the Study of Pain. All efforts were made to minimize animal suffering and to reduce the number of animals used

### 4.2. Pain Model, Behavioral Testing, and Intrathecal Injection

Spinal nerve ligation (SNL)-induced neuropathic pain model—before surgery, we performed the von Frey test and used the animals responding to ≥10-g filaments. The animal neuropathic pain model was constructed by L5 SNL in rats according to a previous study [10]. Briefly, the rat was placed under general anesthesia via inhalation of isoflurane 2% in an oxygen mixture (Hana Pharm Co., Ltd., Daejeon, Korea) and placed in a prone position; the transverse process was carefully removed to visually identify the L4 and L5 spinal nerves. The left L5 spinal nerve was isolated and tightly ligated with 3-0 silk thread (Ethilion, Somerville, NJ, USA). A complete hemostasis was confirmed, and the wound was sutured. A surgical procedure for the sham group was identical to the procedure used for the SNL group, except the spinal nerves were not ligated.

Monoiodoacetate-induced knee osteoarthritis pain model—an osteoarthritic (OA) pain model was established using intra-articular injections of monoiodoacetate (MIA; Sigma, St. Louis, MO, USA) as described previously [35]. Briefly, under isoflurane anesthesia (2% in oxygen mixture; Hana Pharm, Kyung-gi, Korea), saline (25 μL) or MIA (2 mg in 25 μL saline) was delivered to the intra-articular space of the knee joint of the left hind leg through the left patellar tendon using a Hamilton syringe with a 26.5 G needle. After seven days, each solution was administered intrathecally to observe its analgesic effects on OA pain. Afterwards, the animals underwent behavioral tests on the indicated days.

Mechanical threshold test (von Frey filaments test)—the rats were placed on a metal mesh floor covered with clear plastic cages (18 cm × 8 cm × 8 cm) and allowed a 20-min period for habituation. Mechanical stimuli were applied with nine different von Frey filaments ranging from 0.6 to 15-g (0.6, 1, 1.4, 2, 4, 6, 8, 10, and 15 g). Stimuli were applied with a von Frey filament in 3–4-s trials, each of which was repeated four times on each hind paw at approximately 5-min intervals. The 2-g filament stimulus was applied first. If a positive response occurred, the next-smallest von Frey filament was used; if a negative response occurred, the next-largest filament was applied.

Catwalk gait analysis—gait and locomotion in rats were investigated using the CatWalk XT system (Noldus Information Technology, Wageningen, The Netherlands) according to the manufacturer’s instructions [45]. Briefly, rats walked freely on a glass platform along a dark tunnel and fluorescent light was emitted from the platform in proportion to the pressure of each paw. The signal was detected by a video camera under the platform and used for the analysis of gait parameters, such as paw print area and single stance, with CatWalk XT software (Noldus Information Technology, RRID:SCR_004074). The print area was defined as the surface area of the paw in contact with the glass floor. Single stance was defined as the part of the step cycle of the hind paw where the contralateral hind paw did not touch the glass plate or where the ipsilateral hind paw touched the glass plate. The print area and single stance are presented as the percentage of ipsilateral/contralateral.

Intrathecal injection—the intrathecal injection was performed using aseptic techniques according to a previous report [46]. In brief, the injection site was between the last lumbar vertebra and the first sacral vertebrae (L6–S1). Using a Hamilton syringe (50 μL, Reno, NV, USA) with a 26-G hypodermic needle, 10 μL of solution (saline or EB) was administered intrathecally. EB was dissolved in saline. The injection was considered successful if the rat shook its tail during the injection. Animals were divided into sham, SNL plus saline, SNL plus gabapentin (5 mg), and SNL plus EB (5, 15, 50, or 100 µg) groups.

### 4.3. Tissue Processing and Immunohistochemistry

The animals were anesthetized by an intraperitoneal injection of sodium pentobarbital (100 mg/kg) and intracardially perfused with ice-cold 0.05 M phosphate-buffered saline (PBS) containing 0.5% heparin, followed by 4% paraformaldehyde in 0.5 M PBS. Then, the lumbar (L4–5) spinal cords were removed immediately, post-fixed in the same fixative, and cryoprotected with 30% sucrose in 0.05 M PBS. After two days, a cryostat was used to cut the spinal cord segments into 30-μm transverse sections and they were preserved in a storage buffer.

For fluorescent staining, after the incubation of tissues with the blocking buffer (5% normal serum/0.3% Triton X-100) for 1 h to prevent nonspecific binding, the sections were incubated with primary antibodies (Iba-1, 1:400, Wako (Osaka, Japan), 019-19741, RRID:AB_839506; anti-NeuN, 1:100, (Millipore Sigma, MO, USA), MAB377, RRID:AB_2298772; anti-GFAP, 1:1000, Millipore, AB5804, RRID:AB_2109645; anti-VNUT, 1:500, MBL, BMP079, RRID:AB_10597575) diluted in the blocking buffer as reported previously [16,47,48]. Subsequently, the sections were washed with PBS three times and incubated with FITC conjugated-secondary antibody (Jackson ImmunoResearch, West Grove, PA, USA). The sections were counterstained with DAPI (5 μg/mL, Thermo Fisher Scientific, Waltham, MA, USA) and mounted on slides with a mounting medium (Biomeda, Foster, CA, USA). The images were captured by a confocal microscope (Leica, TCS SP8, Wetzlar, Germany) and analyzed with ImageJ software (NIH Image, https://imagej.net/ImageJ, RRID:SCR_003070).

For DAB staining, the tissues were firstly exposed to 0.3% H_2_O_2_ solution for 10 min and incubated with blocking solution. Then, they were treated with a primary antibody (Iba-1, 1:400, Wako) overnight at 4 °C, followed by incubation with biotinylated secondary antibodies and streptavidin peroxidase complex (Vector Laboratories, Inc., Burlingame, CA, USA). The specimens were visualized with diaminobenzidine (DAB, Sigma), and mounted using Polymount (Polysciences, Warrington, PA, USA). The images were obtained on the bright-field microscope ECLIPSE E600 POL (Nikon, Tokyo, Japan).

### 4.4. Reactive Oxygen Species (ROS) Detection Assay

Superoxide anion levels in the spinal cord were determined using dihydroethidium (DHE, Thermo Fisher Scientific), as described previously [49]. Spinal cord sections were incubated with DHE (1 μM) at room temperature for 5 min and mounted on slides. Sections were imaged using a confocal microscope. The fluorescent intensity was quantified with ImageJ software.

### 4.5. Cerebrospinal Fluid (CSF) Collection

The animals were first anesthetized by an intraperitoneal injection of sodium pentobarbital (100 mg/kg). Next, each rat was held by one investigator with the head positioned at a 90° angle. Cerebrospinal fluid (CSF) was obtained by direct perpendicular puncture of the cisterna magna using an insulin syringe (31-G needle, BD). Approximately 100 μL of CSF were collected to avoid blood contamination.

### 4.6. Cell Cultures

The murine microglial cell line (BV2, RRID: CVCL_0182) and hippocampal neuronal cell line (HT22, RRID: CVCL_HT22) were maintained in DMEM supplemented with 10% heat-inactivated fetal bovine serum (FBS) and 1% antibiotics. All cultures were maintained at 37 °C in a 5% CO_2_ humidified incubator.

Primary rat mixed glia cultures were prepared from the cerebral cortices of postnatal day 1–2 Sprague‒Dawley rats, as previously described [50]. After removing the meninges, the cortical tissues were triturated mechanically in DMEM with 10% FBS, passed through a 100-μm nylon mesh cell trainer, and seeded on noncoated plastic dishes or plates in DMEM with 10% FBS and 1% antibiotics, replacing the medium completely every five days. When achieving confluency (day 20‒25), microglia and astrocytes were separated by mild trypsinization (0.0625% trypsin-EDTA in DMEM) for 30 min. While astrocytes were detached from the microglia on top, microglial cells remained at the bottom of the dishes. Isolated astrocytes and microglia were allowed to rest overnight prior to treatments. The purity of microglia or astrocytes was ~97%, examined by immunostaining with Iba1 (microglia marker) or GFAP (astrocytic marker) antibodies. To investigate whether EB was able to disturb ATP release from neurons or glial cells, glutamate-induced ATP release from HT22 cells (mouse neuronal cell line), LPS-induced ATP release from BV2 cells, and glutamate-induced ATP release from rat primary astrocytes were determined in the presence or absence of EB for 2 h (EB, 1 mM; glutamate, 100 µM; LPS, 100 ng/mL). rpm

### 4.7. ATP Assay

The ATP levels in rat CSF and cell culture medium were assessed using the ENLITEN^®^ ATP Assay System Bioluminescence Detection Kit (Promega, Madison, WI, USA) according to the manufacturer’s instructions. Briefly, CSF or culture medium was placed in 1.5-mL Eppendorf tubes and centrifuged at 12,000× g for 5 min at 4 °C to remove debris. Subsequently, 10 μL of the supernatant were mixed with 90 μL of ATP detection solution at its working dilution in a 1.5-mL Eppendorf tube. Luminance (RLU) was measured using a luminometer (Thermo Fisher Scientific; CSP4189), and the concentration of ATP was calculated.

### 4.8. Quantitative Polymerase Chain Reaction (qPCR)

Total RNA was extracted from spinal cord tissues (L4–L5 segment, 0.7 cm) or cells using TRIzol reagent (Invitrogen, Carlsbad, CA, USA) according to the manufacturer’s instructions. After purification with a RNA isolation kit (Hybrid-R, GeneAll Biotechnology, Seoul, Korea, 305-101), the concentration and purity of RNA were assessed using the NanoDrop spectrophotometer.

Total RNA (4 µg) was used for cDNA synthesis with a TOPscript cDNA synthesis kit (Enzynomics, Daejeon, Korea; RT220) in a 20 µL reaction. Subsequently, quantitative polymerase chain reaction (qPCR) was performed in duplicate in a total volume of 10 µL including 2 µL of each 5 pM primer, 4 µL of cDNA, and 5 µL of TOPreal qPCR SYBR mix (Enzynomics, RT500). The qPCR conditions were 95 °C for 10 min, then 40 amplification cycles of 95 °C for 15 s and 60 °C for 1 min (AriaMx Realtime PCR System, Agilent Technologies, Santa Clara, CA, USA). The primer sequences used for the amplifications were as follows: VNUT, forward: 5′-GCTTCATCACTGTCACCACA-3′, reverse: 5′-CCAGGACAAGGTCTTTCTCA-3′; GAPDH, forward: 5′-CTCATGACCACAGTCCATGC-3′, reverse: 5′-TTCAGCTCTGGGATGACCTT-3′; IL-1β, forward: 5′-CAGCAGCATCTCGACAAGAG-3′, reverse: 5′-CATCATCCCACGAGTCACAG-3′; TNF-a, forward: 5′-AGATGTGGAACTGGCAGAGG-3′, reverse: 5′-CCCATTTGGGAACTTCTCCT-3′; IL-6, forward: 5′-CCGGAGAGGAGACTTCACAG-3′, reverse: 5′-ACAGTGCATCATCGCTGTTC-3′; iNOS forward: 5′-TCTGTGCCTTTGCTCATGACA-3′, reverse: 5′-TGCTTCGAACATCGAACGTC-3′; COX-2 forward: 5′-CAGTATCAGAACCGCATTGCC-3′, reverse: 5′-GAGCAAGTCCGTGTTCAAGGA-3′. The mRNA expression level was normalized to GAPDH, and the relative mRNA expression was calculated using the 2^−ΔΔ*C*t^ method, as previously described [51].

### 4.9. Statistical Analysis

The data are expressed as mean  ±  standard error of the mean (SEM). The statistical significance of differences among multiple groups was calculated using one-way or two-way analysis of variance (ANOVA), followed by an appropriate multiple comparison test. Two-group analyses were performed using the two-tailed unpaired Student’s *t*-test. *p*-values  <  0.05 were considered statistically significant. All statistical analyses were performed using GraphPad Prism 6 software (GraphPad Software, La Jolla, CA, USA; RRID:SCR_002798).

## Abbreviations

VNUT	Vesicular nucleotide transporter
EB	Evans blue
SNL	Spinal nerve ligation
IL	Interleukin
qPCR	Quantitative polymerase chain reaction (qPCR)
CSF	Cerebrospinal fluid
MIA	Monoiodoacetate
OA	Osteoarthritis
